# Analysis of liver iron concentration in an elderly female undergoing hemodialysis with calcific uremic arteriolopathy does not support the role of iron overload in calciphylaxis: lesson for the clinical nephrologist

**DOI:** 10.1007/s40620-020-00904-z

**Published:** 2021-01-23

**Authors:** Guy Rostoker, Patricia Senet, Fanny Lepeytre, Mireille Griuncelli, Christelle Loridon, Clémentine Rabaté, Yves Cohen

**Affiliations:** 1 Division of Nephrology and Dialysis, Ramsay Santé, Hôpital Privé Claude Galien, 20 Route de Boussy-Saint-Antoine, 91480 Quincy-Sous-Sénart, France; 2 Collège de Médecine des Hôpitaux de Paris, 10 Rue des Fossés Saint-Marcel, 75005 Paris, France; 3 Division of Dermatology and Allergology, Assistance Publique Hôpitaux de Paris, Hôpital Tenon, Sorbonne Université, 4 Rue de la Chine, 75020 Paris, France; 4 Division of Radiology, Ramsay Santé, Hôpital Privé Claude Galien, 20 Route de Boussy-Saint-Antoine, 91480 Quincy-Sous-Sénart, France; 5 Service de Néphrologie et de Dialyse, Ramsay Santé, HP Claude Galien, 20 Route de Boussy-Saint-Antoine, 91480 Quincy-Sous-Sénart, France

**Keywords:** Calciphylaxis, Iron overload, Liver iron concentration, MRI

## Case

An 88-year-old female patient started hemodialysis in August 2016 using a tunneled catheter due to end-stage kidney disease (ESKD) related to idiopathic membranous nephropathy combined with diabetic nephropathy (Table 1). She had suffered from type 2 diabetes mellitus since the age of 54 years (complicated by mild diabetic retinopathy and mild sensitive polyneuritis of the lower limbs), had a long history of diastolic cardiac insufficiency and had suffered from atrial fibrillation since 2016 with high risk of thromboembolic events, requiring long-term anticoagulation with fluindione (a vitamin K antagonist) (VKA). She was considered to have very good physiologic and psychological adaptation to hemodialysis despite her old age.

**Table 1:** Demographic and clinical characteristics of our elderly dialysis patient with calciphylaxis, studied by MRI to determine liver and spleen iron content

Characteristics	
Age (years)	88
Dialysis duration (months)	35.9
Mean monthly iron dose since initiation of dialysis (mg)	198
Mean monthly darbepoietin dose (μg) since initiation of dialysis	172
Body Mass Index (kg m^−2^)	26.3
Glycated hemoglobin (%)	June 2018: 7.5 (Hb: 11.4)
(Absolute hemoglobin level (g/dl) at the time of HbA1c dosage)	June 2019: 8.2 (Hb: 11.5)
Albumin (g/l)	32
Prealbumin (g/l)	0.19
*LIC by Signal intensity ratio MRI according to Rennes University (μmol/g)* (Normal value ≤ 50 μmol/g)	62
LIC by classical R2* MRI	
Value by T2* expressed in ms (normal value > 15 ms)	11.7
Value of LIC using Garbowski equation (μmol/g) (normal value < 36 μmol/g)	47.23
LIC by R2* IDEAL-IQ MRI	
Value by T2* IDEAL-IQ expressed in ms (normal value > 15 ms)	11.1
Value of LIC using Garbowski equation (μmol/g) (normal value < 36 μmol/g)	49.82
SIC by classical R2* MRI	
Value by T2* expressed in ms (normal value > 15 ms)	17.9
Value of SIC using Garbowski equation (μmol/g) (normal value < 36 μmol/g)	30.69

*LIC* liver iron concentration, *SIC* spleen iron concentration

**Fig. 1:**
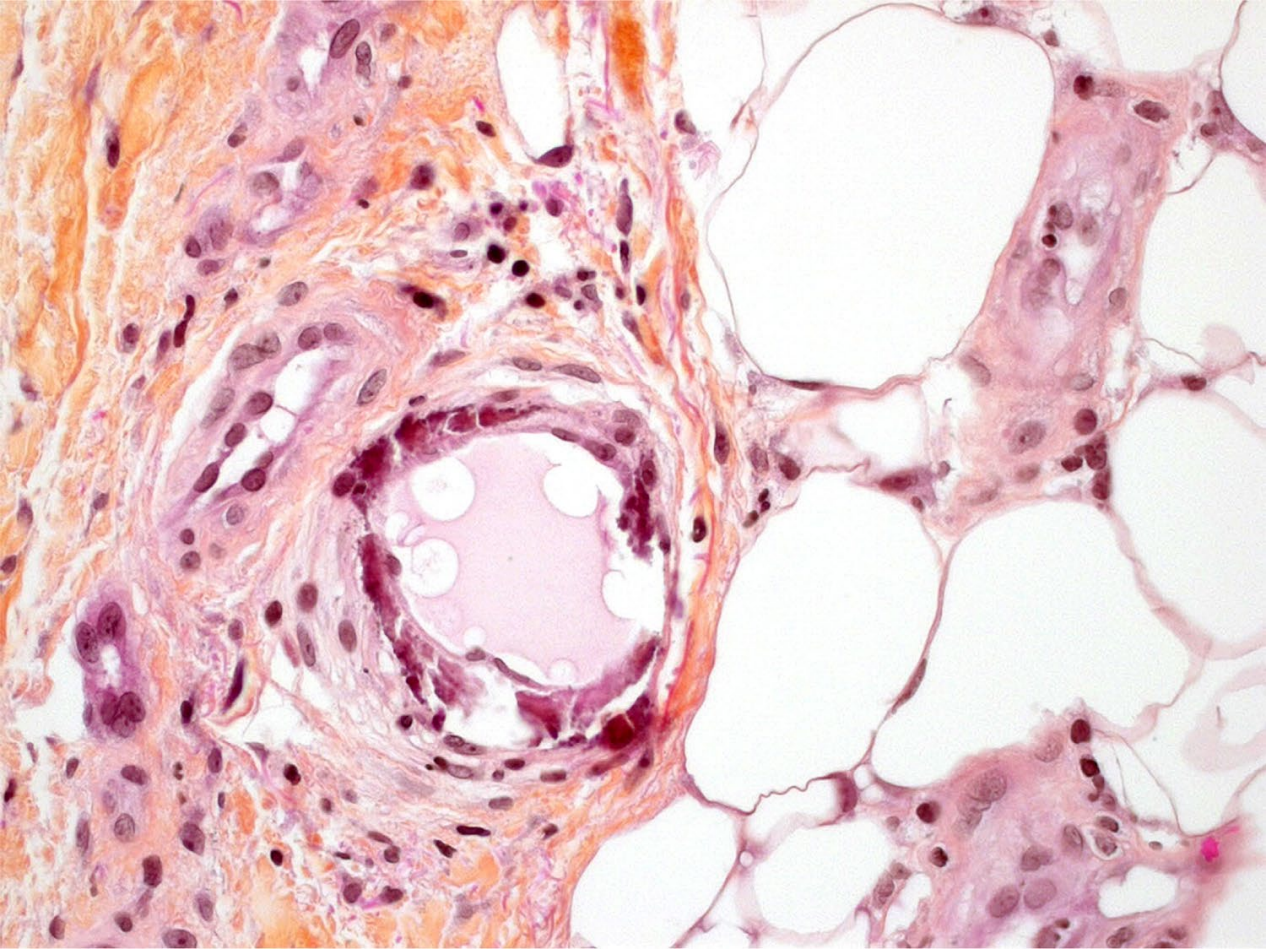
Photography of the skin biopsy of the case at hematoxylin-eosin-saffron staining, magnification 400, showing massive circumferential calcification of a hypodermic capillary

Apart from the failure of vascular access surgery, she had no dialysis or general complications until June 2019, when extensive painful ulcers appeared on both legs without any evidence of peripheral arterial disease on imaging (vascular computerized axial tomography coupled with ultrasound scans); the ulcers were preceded by erysipelas-like lesions. The patient was referred for a dermatology consultation to the Tenon hospital, Paris, where calciphylaxis was clinically suspected and formally diagnosed on a deep dermal biopsy (16th July, 2020) (Fig.1). Serum calcium, phosphorus, and aluminum were within the normal range, whereas parathyroid hormone and bone alkaline phosphatase levels were slightly low for a dialysis patient (Table 2). Serum iron biomarkers were close to the target of the European statement for anemia management [1] (Table 2). The patient underwent multidisciplinary management with hospitalization in a geriatric unit (from 23rd August, 2019 to 25th March, 2020) with pain relief by opioids and pregabalin, daily wound cleaning by specialized nurses, together with intensification of dialysis by Expanded Hemodialysis (HDx therapy with high cut-off dialyzer Theranova^®^) 3-times a week for 4 h (because of the patient’s refusal of daily dialysis) with administration of sodium thiosulfate (25 g infused during the last hour of each dialysis session until 13th December, 2019, without any side-effects during the whole duration of therapy). The disappearance of lower limb pain occurred in November 2019 and opioids and pregabalin were therefore stopped, respectively, 12th November and 18th November, 2019.

**Table 2:** Biochemical markers of bone and iron metabolism in a dialysis patient with calciphylaxis, studied by MRI to determine liver iron content

Biochemical marker	Concentration
*Serum calcium* Complexometry colorimetric technic (Roche diagnostics) (Normal range: 2.20–2.55 mmol/L)	2.05 mmol/L
*Corrected serum calcium* (Normal range: 2.20–2.55 mmol/L)	2.39 mmol/L
*Serum phosphate* Colorimetric technic (Roche diagnostics) (normal range: 0.81–1.45 mmol/L)	1.62 mmol/L
*Serum aluminium* Atomic absorption (Normal value in dialysis patient < 20 µg/L*)*	3 µg/L
*Serum parathyroid hormone* Chemiluminescence (Normal range in non-renal patients: 15–65 pg/mL)	151 pg/mL
*Serum bone alkaline phosphatase* Chemiluminescence (Normal range in premenopausal women: 4.9–26.6 µg/L) (Normal range in postmenopausal women: 5.2–24.4 µg/L)	3.8 µg/L
*Serum ferritin* Chemiluminescence (Alinity, Abbott) (Normal range: males: 22–275 µg/L, females:15–204 µg/L)	256 µg/L
*Serum transferrin saturation* Serum iron/total iron-binding capacity ratio (Normal range: 15–35%)	18%
*Serum C-reactive protein* Immunoturbidimetry (Roche Diagnostics) (Normal value: < 5 mg/L)	36.2 mg/L

The vitamin K antagonist was discontinued on 24th July, 2019 as it was considered to be responsible for calciphylaxis as a drug-related adverse event [2]; calcium dialysate (1.5 mmol) remained unchanged.

Our cardiology team advised us to pursue general anticoagulation given her high risk of thromboembolic events. Danaparoid was used (for both general and dialysis), guided by measurement of residual anti-Xa danaparoid. Danaparoid was stopped on 28th January, 2020 and replaced with 10 mg oral rivaroxaban daily and 2000 IU heparin during her dialysis sessions.

The patient underwent two successful split-thickness skin grafts on 14th and 28th October, 2019 in the dermatology unit of Tenon hospital. A dermatology consultation on 10th December, 2019 showed 90% healing of leg ulcers, and sodium thiosulfate was therefore stopped. A subsequent dermatology consultation on 10th March, 2020 confirmed complete healing of the leg ulcers and she was discharged home at the end of March 2020.

Liver iron concentration (LIC) was measured in this patient on 13th August, 2019 by quantitative magnetic resonance imaging (qMRI) comprising three simultaneous methods: Signal Intensity Ratio (SIR) according to Rennes University, classical R2* relaxometry, and R2* relaxometry with multi-peak fat spectral modeling (R2*-IDEAL-IQ relaxometry) [3]. The Garbowski equation was used in R2* relaxometry (classical and IDEAL-IQ) to translate hepatic T2* expressed in ms into µmol/g dry liver [4].

Splenic iron concentration (SIC) was also assessed during the same session by R2* relaxometry [3] and SIC concentration was extrapolated in µmol/g using the Garbowski equation [4].

The LIC of our patient was slightly increased (62 µmol/g dry weight) on SIR-MRI (normal values $\le$ 50 µmol/g) with similar findings on R2* relaxometry (classical and IDEAL-IQ) [3] (Table 1), whereas her SIC was normal (30.69 µmol/g; normal < 36 µmol/g) (Table 1). Iron therapy, which was decreased in mid-July (100 mg/month intravenous (i.v.) iron sucrose) when the diagnosis of calcific uremic arteriolopathy (CUA) was established, was then continued at the same dose after the MRI examination (100 mg i.v. iron sucrose given systematically twice a month).

## Lesson for the clinical nephrologist

Calcific uremic arteriolopathy (also named calciphylaxis) is a rare and devastating disorder predominantly affecting dialysis patients, It is favored by diabetes mellitus, obesity, high levels of serum calcium, phosphorus and parathyroid hormone, and also by vitamin K antagonist treatment; mean CUA rate is about 3.49 per 1000 patient-years and mortality rates of up to 50% have been described at 12 months [2] (Supplemental material ref 1).

The finding of significant amounts of iron in the skin lesions of 12 patients with CUA in the USA by mass spectrometry but lacking in controls [5], as well as the histologic visualization of iron deposits in areas of microvascular calcifications in 12 Canadian patients with CUA [6], suggest a potential role of iron in the pathophysiology of calciphylaxis. This hypothesis was fueled by the pioneering experimental studies of Selye et al*.* who investigated the postulated two-hit pathophysiology of calciphylaxis using ferric dextran (Supplemental material ref 2), and cases of CUA in hemodialysis patients with transfusional hemosiderosis in the pre-epoetin era (Supplemental material ref 3). Furthermore, the preliminary results of the DOPPS study suggested that infused iron concentrations > 400 mg/month might also be associated with CUA (Supplemental material ref 4).

The liver is the main site of iron storage and liver iron concentration is a valuable marker of total body iron stores which can be measured non-invasively and accurately by quantitative magnetic resonance imaging in iron overload disorders, including in dialysis patients [3].

For the past two decades, erythropoiesis-stimulating agents and intravenous iron have been the backbone of anemia treatment in end-stage kidney disease, owing to the decreased release of epoetin by diseased kidneys and iron deficiency due to blood loss related to the hemodialysis procedure and to uremic enteropathy, the latter condition being aggravated by functional iron deficiency (with iron trapping in the reticuloendothelial system) related to high hepcidin levels encountered in ESKD [1].

Recent studies of liver iron stores in dialysis patients by qMRI and susceptometry have revealed a high frequency of radiologic hemosiderosis (affecting up to 66% of 500 dialysis patients in a pooled analysis of 11 published radiologic studies on LIC (susceptometry = 1; MRI = 10)), therefore questioning the potential hazards of routine, indiscriminate administration of high dose i.v. iron in this setting (Supplemental material ref 5, 6, 7).

The relationship between vitamin K antagonists and skin lesions is not simple in ESKD because the clinical aspect of calciphylaxis may evoke skin necroses induced by VKA. However, the context of VKA-induced skin necroses strongly differs from that of calciphylaxis since they are due to a transient hypercoagulable state usually occurring at the initiation of vitamin K antagonist treatment (related to an imbalance between pro and anticoagulant factors). However, rare observations of atypical chronic leg ulcers induced by long term prescription of VKA have been described but with skin histology showing either neutrophilic dermatoses, vasculitis or micro-thrombosis.

Here, we analyzed the liver iron stores by qMRI in a typical case of CUA occurring in an elderly female diabetic hemodialysis patient and observed a slight increase in her LIC. This observation prompted us to investigate the occurrence of other cases of calciphylaxis in a prospective, cross-sectional and longitudinal study which was started on 31st January, 2005 in the department of nephrology and dialysis of the Claude Galien hospital (France) and which is still ongoing. Its aim was to study iron stores in dialysis patients by qMRI and its determinants. The study is registered under International Standard Randomized Controlled Trial Number (ISRCTN): 80100088. Details of this study have been published previously (Supplemental material ref 5). Our research group has now analyzed LIC by qMRI in 358 dialysis patients (from January 2005 to February 2020), of whom 115 had normal liver iron (LIC $\le$ 50 µmol/g dry liver on SIR-MRI) and 243 had increased LIC: 114 patients had mild iron overload (50 < LIC $\le$ 100 µmol/g on SIR-MRI), 58 had moderate iron overload (100 < LIC $\le$ 200 µmol/g on SIR-MRI), and 71 had severe iron overload (> 200 µmol/g dry liver on SIR-MRI). Analysis of the medical charts of the 243 dialysis patients with increased LIC showed that the patient presented in the case was the only one with CUA. Thus, none of the others with mild (*n* = 113), moderate (*n* = 58), or severe (*n* = 71) radiologic iron overload had calciphylaxis; no case of CUA was observed in any dialysis patient with normal LIC (*n* = 115).

Very small increases in LIC have been found in heterozygous patients with hereditary hemochromatosis without any clinical consequences [7], and this very low level of iron overload is considered safe in thalassemic patients receiving chelation therapy providing they have a normal cardiac MRI [8]. Similarly, dialysis patients with a small increase in LIC did not have an increased risk of induced or aggravated non-alcoholic fatty liver disease in contrast to those with moderate or severe iron overload [9].

This case also highlights the interest of qMRI for monitoring iron stores in dialysis patients, especially those with CUA, owing to the influence of inflammation on iron biomarkers, making them inadequate for guiding iron repletion (Supplemental material ref 5 and 8). Conversely, our case and the analysis of our larger study cohort do not support the recent expert opinion to stop i.v. iron administration in patients with CUA [2], but rather argue for close monitoring of iron therapy based on iron store analysis by MRI.

## Electronic supplementary material

Below is the link to the electronic supplementary material.

Supplementary file1 (DOCX 18 KB)
