# Supplementary material for: Analysis of liver iron concentration in an elderly female undergoing hemodialysis with calcific uremic arteriolopathy does not support the role of iron overload in calciphylaxis: lesson for the clinical nephrologist
Source: J Nephrol. 2021 Jan 23;34(5):1547–51. doi: 10.1007/s40620-020-00904-z (PMC8494698; doi:10.1007/s40620-020-00904-z)
Supplement: Supplementary file 1 — Supplementary file1 (DOCX 18 KB) [file 40620_2020_904_MOESM1_ESM.docx]

**Supplemental material**

*Additional references*

1. Nigwekar SU, Zhao S, Wenger J, et al. A nationally representative study of calcific uremic arteriolopathy risk factors. J Am Soc Nephrol. 2016;27(11):3421-3429.
2. Selye H, Gabbiani G, Tuchweber B. Calciphylaxis: passive transfer. Science.1964;143(3604):365-366.
3. Rubinger D, Friedlaender MM, Silver J, et al. Progressive vascular calcification with necrosis of extremities in hemodialysis patients: a possible role of iron overload. Am J Kidney Dis. 1986;7(2):125-129.
4. Goodkin DA, Larkina M, Robinson BM. Iron and calciphylaxis. Nephrol Dial Transplant. 2011;26(9):3063; author reply 3063-3064.
5. Rostoker G, Griuncelli M, Loridon C, et al. Hemodialysis-associated hemosiderosis in the era of erythropoiesis-stimulating agents: a MRI study. Am J Med. 2012;125(10):991‒999.
6. Vaziri ND. Epidemic of iron overload in dialysis population caused by intravenous iron products: a plea for moderation. Am J Med. 2012;125(10): 951-952.
7. Rostoker G, Vaziri ND. Risk of iron overload with chronic indiscriminate use of intravenous iron products in ESRD and IBD populations. Heliyon. 2019;5(7):e02045.
8. Ferrari P, Kulkarni H, Dheda S, et al. Serum iron markers are inadequate for guiding iron repletion in chronic kidney disease. Clin J Am Soc Nephrol. 2011;6(1):77-83.
